# Development and characterization of near-isogenic lines for brown planthopper resistance genes in the genetic background of *japonica* rice ‘Sagabiyori’

**DOI:** 10.1270/jsbbs.23017

**Published:** 2023-09-09

**Authors:** Saw Bo Day Shar, Cuong Dinh Nguyen, Sachiyo Sanada-Morimura, Hideshi Yasui, Shao-Hui Zheng, Daisuke Fujita

**Affiliations:** 1 The United Graduate School of Agricultural Sciences, Kagoshima University, 1-21-24 Korimoto, Kagoshima 890-0065, Japan; 2 Loikaw Research Center, Department of Agricultural Research, Loikaw 09011, Kayah State, Myanmar; 3 Biotechnology Department, College of Food Industry, 101B Le Huu Trac Street, Son Tra District, Da Nang City 550000, Vietnam; 4 Agro-Environment Research Division, Kyushu Okinawa Agricultural Research Center, NARO, 2421 Suya, Koshi, Kumamoto 861-1192, Japan; 5 Plant Breeding Laboratory, Graduate School, Kyushu University, Fukuoka 812-8581, Japan; 6 Faculty of Agriculture, Saga University, 1 Honjo-machi, Saga 840-8502, Japan

**Keywords:** marker-assisted selection, *Nilaparvata lugens*, BPH resistance gene, NILs

## Abstract

The brown planthopper (BPH: *Nilaparvata lugens* Stål) is one of the most destructive insects in rice production. The use of host plant resistance has potential to reduce damage caused by BPH. The heat tolerance *japonica* rice ‘Sagabiyori’, with superior grain quality and high soluble starch in the stem, is highly susceptible to damage by BPH. Here, to enhance its BPH resistance, we developed seven near-isogenic lines (NILs) carrying *BPH2*, *BPH17-ptb*, *BPH32*, *BPH3*, *BPH17*, *BPH20*, and *BPH21* through marker-assisted selection and evaluated resistance to two BPH populations. Most lines were more resistant to the Hadano-1966 BPH population than Sagabiyori but were less effective against the highly virulent Koshi-2013 population. Nevertheless, in antixenosis tests, Koshi-2013 settled less on all NILs than on Sagabiyori. In addition, adult mortality and the percentage of fresh weight loss of lines carrying *BPH17* and *BPH3* indicated that these lines have higher resistance to Koshi-2013 than Sagabiyori. Current study revealed that BPH resistance of Sagabiyori became stronger by transferring *BPH3* and *BPH17* genes. Thus, *BPH3* and *BPH17* might be valuable for breeding programs to enhance BPH resistance of high grain quality rice varieties with heat tolerance.

## Introduction

Rice (*Oryza sativa* L.) is one of the world’s most important food crops, feeding more than half of the population. The brown planthopper (BPH), *Nilaparvata lugens* Stål, is a destructive pest that directly sucking phloem sap and vectors *Rice grassy stunt virus* (RGSV) and *Rice ragged stunt virus* (RRSV) diseases (Bottrell and Schoenly 2012). Heavy infestation by BPH results in the complete death of rice plants. In East Asia, BPH migrates from northern Vietnam to southern China and is carried from China to Japan by strong southwesterly winds during the rainy season (Hu *et al.* 2017). Between 2005 and 2008, a total of 2.7 million t of rice yield was lost over four years in China (Hu *et al.* 2016a) due to direct damage from BPH, while 0.4 million t was lost in Vietnam, mainly to RGSV and RRSV (Brar *et al.* 2009). Furthermore, in 2013, there was outbreaks of BPH occurred in western and southwestern Japan, resulting in a loss of 10.5 billion yen in rice production. In addition, outbreaks of BPH in 2019 caused almost the same tremendous economic losses to Japanese rice farmers as in 2013 (Sanada-Morimura 2020).

The improvement of host plant resistance in rice is used to reduce BPH damage (Dyck and Thomas 1979, Khush 1995) and is considered one of the most cost-effective and environmentally friendly strategies for BPH management. To date, more than 45 loci for BPH resistance (designated *BPH1* to *BPH45*) have been identified in rice (Du *et al.* 2020, Wang *et al.* 2022, Yang *et al.* 2019, Zhang *et al.* 2020). Among genes mapped to specific locations, most are clustered on chromosomes 12L (cluster A), 4S (cluster B), 6S (cluster C), 4L (cluster D), and 3L (cluster E) (Fujita *et al.* 2013, Hu *et al.* 2016a). Among these, nine genes *BPH6*, *BPH9*, *BPH14*, *BPH17*, *BPH18*, *BPH26*, *BPH29*, *BPH32*, and *BPH30* have been cloned and characterized for BPH resistance (Du *et al.* 2009, Guo *et al.* 2018, Ji *et al.* 2016, Ren *et al.* 2016, Shi *et al.* 2021, Tamura *et al.* 2014, Wang *et al.* 2015, Zhao *et al.* 2016).

Monogenic resistance is vulnerable to rapid adaptation by pest populations. BPH populations have sufficient genetic variability to enable them to overcome specific resistance genes in a resistant cultivar over several generations (Zhao *et al.* 2016). In the late 1970s, BPH populations adapted to cultivars with the *BPH1* and *BPH2* genes (IR26, IR36, etc.), after these cultivars were widely adopted across Asia (Saxena and Barrion 1985). In addition, continuous rearing of BPH populations on resistant rice cultivars for 7 to 15 generations under controlled conditions allowed adaptation to resistance to *BPH1*, *BPH2*, *BPH3*, *BPH8*, *BPH9*, *BPH10*, and *BPH32* (Claridge and Den Hollander 1982, Ferrater *et al.* 2015, Ketipearachchi *et al.* 1998). A recent multinational study indicated that BPH populations across Asia have adapted to rice cultivars carrying *BPH1*, *BPH2*, *BPH5*, *BPH7*, *BPH8*, *BPH9*, *BPH10*, and *BPH18* (Horgan *et al.* 2015). In long-term monitoring of BPH from 2001 to 2019 in Japan, the survival rates of immigrant populations of BPH collected during 2016–2018 on the highly resistant ‘Rathu Heenati’ and ‘Balamawee’ were 11%–20% and 22%–64%, respectively (Fujii *et al.* 2021). Natural BPH populations are becoming progressively more virulent to resistant cultivars through adaptation.

The effects of any single resistance gene may be revealed in detail by using near-isogenic lines (NILs) that carry the gene in the genetic background of a susceptible cultivar. At least 16 NILs with BPH resistance genes (*BPH3*, *BPH4*, *BPH6*, *BPH9*, *BPH10*, *BPH12*, *BPH14*, *BPH15*, *BPH17*, *BPH18*, *BPH20*, *BPH21*, *BPH25*, *BPH26*, *BPH30*, and *BPH32*) have been developed in the genetic backgrounds of several susceptible *indica* and *japonica* cultivars (Hu *et al.* 2016b, Jena *et al.* 2017, Myint *et al.* 2012, Nguyen *et al.* 2019, Qiu *et al.* 2012, Shi *et al.* 2021). The resistance of cultivars with single genes is weaker and less durable than that of cultivars with multiple resistance genes. Researchers have proposed the pyramiding of two or more genes to enhance resistance and thereby avoid pest adaptation (Horgan *et al.* 2015). Combinations of multiple BPH resistance genes have increased resistance to BPH. For instance, a pyramided line (PYL) with *BPH14* and *BPH15* had greater resistance to BPH from China than NILs with either gene alone (Hu *et al.* 2016a). Similarly, pyramiding of *BPH25* and *BPH26* had positive epistatic effects against BPH populations collected in Vietnam, the Philippines, and Japan (Myint *et al.* 2012). Therefore, the development of cultivars carrying multiple BPH resistance genes might be an effective way to enhance BPH resistance.

The *japonica* cultivar ‘Sagabiyori’ has a “special A” rating for its high eating quality and grain quality, and is grown in more than 20% of rice fields in Saga Prefecture, Japan. Moreover, Sagabiyori has heat tolerance: high grain quality under more than 30°C at maturity stage, due to high content of nonstructural carbohydrate (NSC) in the stem as soluble sugars and starch (Tanamachi *et al.* 2016). As a phloem sucking insect, BPH which takes sucrose from leaf sheath, consequently reduce NSC accumulation in the stem and disrupts translocation of assimilate may eventually lead to hopper burn (Watanabe and Kitagawa 2000). Sagabiyori is susceptible to damage from BPH, which is presumably related to its high NSC content. It is necessary to enhance BPH resistance in Sagabiyori with high NSC content but we did not know effectiveness of BPH resistance genes in Sagabiyori. Therefore, this study was conducted to identify factors that inhibit BPH multiplication in existing BPH-resistant genetic resources under Sagabiyori genetic background. Here, we developed seven NILs with BPH resistance genes (*BPH2*, *BPH3*, *BPH17-ptb*, *BPH32*, *BPH17*, *BPH20*, and *BPH21*) in the Sagabiyori genetic background to evaluate the effects of each gene against two BPH populations collected in Japan, one in 1966 (before resistant cultivars were widely released) and one in 2013 (recently arrived from China). To understand factors for suppressing BPH multiply in Sagabiyori background, the NILs for BPH resistance with Sagabiyori background were evaluated detailed.

## Materials and Methods

### Plant materials

**Fig. 1.**
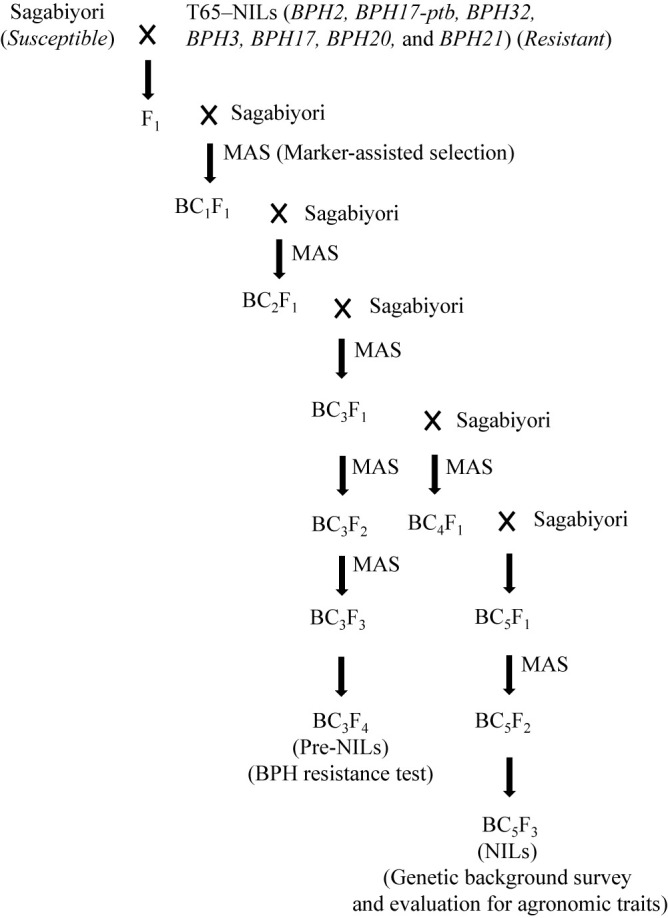
Breeding scheme for development of pre-NILs and NILs carrying BPH resistance genes in the Sagabiyori genetic background.

To develop NILs carrying BPH resistance genes in the Sagabiyori background, we used seven NILs with BPH resistance genes in the ‘Taichung 65’ (T65) genetic background as donor parents (Nguyen *et al.* 2019). *BPH2*, *BPH17-ptb*, and *BPH32* were derived from the broad-spectrum BPH-resistant cultivar ‘PTB33’ (acc. no. IRGC19325) (Angeles *et al.* 1986, Nguyen *et al.* 2021); *BPH3* and *BPH17* from Rathu Heenati (acc. no. IRGC11730) (Jairin *et al.* 2007a, Sun *et al.* 2005); and *BPH20* and *BPH21* from ‘IR-71033-121-15’, which was derived from *Oryza minuta* (Rahman *et al.* 2009). Sagabiyori was crossed with each T65-NIL, and three backcrosses to Sagabiyori generated BC_3_F_1_ plants (Fig. 1). In each backcross generation, plants carrying the BPH resistance genes from the donor parent were selected by marker-assisted selection (MAS) using simple sequence repeat (SSR) markers linked to the target genes (Supplemental Table 1). Self-pollination produced BC_3_F_2_ and BC_3_F_3_ plants with resistance genes. Finally, seven BC_3_F_4_ Saga-*BPH* pre-NILs were developed, one per gene (Table 1). Further backcrossing to Sagabiyori generated BC_5_F_1_ plants, which were self-pollinated to produce BC_5_F_2_ lines as advanced Saga-*BPH* NILs (Table 1). In this study, the lines with BC_5_ generation were denoted as NILs because most of genetic background excepting target gene region from donor was theoretically substituted by recurrent parent. The lines with BC_3_ generation, those were lower background recovery rate of Sagabiyori, designated as pre-NILs. BPH resistance was evaluated in the pre-NILs, and the genetic background was surveyed and agronomic traits were evaluated in the NILs.

### MAS for BPH resistance genes

We collected ~2 cm of leaves of 14-day-old seedlings and freeze-dried them for 48 h. Total DNA was extracted by the potassium acetate method (Dellaporta *et al.* 1983). The genotypes of SSR markers in plants in each generation were determined by polymerase chain reaction (PCR) and electrophoresis. The PCR amplification mix (8 μL) contained 3 μL of 2× GoTaq Green Master Mix (pH 8.5), 1 μL of 0.25 μM primers, and 4 μL of 1:20-diluted DNA. PCR amplification comprised an initial 96°C for 5 min; 35 cycles at 96°C for 30 s, 55°C for 30 s, and 72°C for 30 s; and a final step at 25°C for 1 min. PCR products were separated by electrophoresis at 200 V in 4% agarose gel with 0.5 μg/mL ethidium bromide in 0.5× TBE buffer for 1 h and photographed under ultraviolet light.

For MAS of BPH resistance genes on chromosome (Chr.) 4S, plants with *BPH17* and *BPH17-ptb* were selected using markers RM1305 and B40, and plants with *BPH20* were selected using RM1305 and RM16531. For MAS of genes on the short arm of Chr. 6, plants with *BPH3* and *BPH32* were selected using markers RM508 and RM588. For MAS of genes on the long arm of Chr. 12, plants with *BPH2* and *BPH21* were selected using markers RM28404 and RM28493 (Supplemental Table 1).

**Fig. 2.**
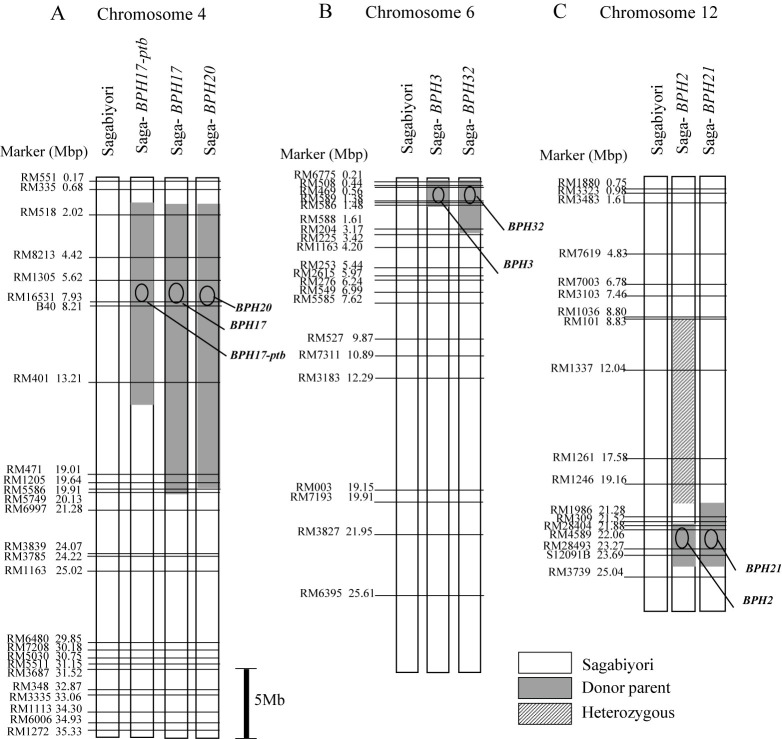
Graphical genotypes of NILs for BPH resistance genes in Sagabiyori genetic background. A. NILs for *BPH17-ptb*, *BPH17*, and *BPH20* on chromosome 4. B. NILs for *BPH3* and *BPH32* on chromosome 6. C. NILs for *BPH2* and *BPH21* on chromosome 12. The map position of each marker is based on physical distance. Circles indicate the approximate position of resistance genes.

To confirm the substitution of chromosomal segments around resistance genes in each NIL, we investigated the genotypes using 65 SSR markers distributed on target segments carrying Chrs. 4, 6, and 12. 26 markers on Chr. 4, 21 on Chr. 6, and 18 on Chr. 12 (Fig. 2). The graphical genotype of each NIL was determined by the polymorphism between Sagabiyori and each NIL in the SSR marker regions using the method of Young and Tanksley (1989). A map was constructed in GGT v. 2.0 software (Van Berloo 2008, Young and Tanksley 1989).

### Genetic background survey of NILs

We surveyed whole genome background of each NIL using genotyping by random amplicon sequencing-direct (GRAS-Di) technology. Total DNA of Sagabiyori and each NIL were extracted from young seedlings by DNeasy Plant MiniKit (Qiagen, Germany). GRAS-Di libraries were constructed (Hosoya *et al.* 2019) and sequencing of the libraries was performed using NovaSeq6000 by Eurofins Genomics, Tokyo. Genotyping was performed by GRAS-Di software (TOYOTA, Aichi, Japan) which are commercially available. The marker quality that was empirically determined based on the number of reads and reproducibility of genotyping were evaluated by GRAS-Di software and the GRAS-Di software ranked markers using A, B, C, D, and E in descending order of quality. Markers of qualities E-ranked was less reliable than those of qualities A, B, C, and D. The quality A, B, C, and D among the GRAS-Di markers were used for estimating substituted chromosomal segments from donor parents. Among GRAS-Di markers, polymorphic and co-dominant markers between each NIL and Sagabiyori were selected to estimate locations of substituted chromosomal segments from donor parents (Supplemental Fig. 1).

### BPH populations used for resistance tests

We used two BPH populations to evaluate the BC_3_F_4_ Saga-*BPH* pre-NILs for resistance. The Hadano-66 population was collected in Hadano City, Kanagawa Prefecture, in 1966 (Myint *et al.* 2009b). This population, which has weakest virulence to rice plant, is crucial for evaluating the effect any BPH resistance gene because it was collected before releasing BPH resistance varieties. Second, Koshi-2013 population was collected in Koshi City, Kumamoto Prefecture, in 2013 (Fujii *et al.* 2021). Both strains were maintained on the susceptible *japonica* cultivar ‘Reiho’ at 25°C under 16 h light/8 h dark at National Agriculture and Food Research Organization (NARO), JAPAN. Both were maintained on T65 under the same conditions at Saga University.

### Modified seedbox screening test

The resistance of all pre-NILs and Sagabiyori to each BPH strain was evaluated by the modified seedbox screening test at 25°C (Horgan *et al.* 2015). We sowed 30 seeds of each line and Sagabiyori in single rows in a plastic tray (23.0 cm × 30.0 cm × 2.5 cm) with 2.5 cm between rows, with three replicates. At 7 days after sowing (DAS), the plants were thinned to 20 plants per row. The plants were then infested by second- and third-instar nymphs at a density of ~20 BPH per plant. When all plants of Sagabiyori were dead, all lines were scored for damage on a scale of 0 to 9 following the standard damage score (DS) evaluation system for rice (IRRI 2014).

### Antibiosis test

Antibiosis tests were conducted at 25°C as described by Myint *et al.* (2009b). Ten seeds of each pre-NIL, Sagabiyori (susceptible control), and Rathu Heenati (resistant control) were individually sown in 215-mL plastic cups. At 4 weeks after sowing, plants were trimmed to 15 cm height and covered with a plastic tube with windows for ventilation. Each tubes were infested by five small-abdomen brachypterous female BPHs. At 5 days after infestation (DAI), the adult mortality (ADM) were calculated as the rate of dead BPH among infested BPH.

The feeding rates of BPH on the pre-NILs were determined as described by Heinrichs *et al.* (1985) with minor modifications. Ten seeds of each pre-NIL and the parents were individually sown in 215-mL plastic cups. When plants were 30 days old, the plants in each cup were covered with an inverted transparent plastic cup with ventilators for keeping insects at the base of plants. A filter paper treated with 0.1% bromocresol green in ethanol was placed inside the inverted transparent plastic cups to absorb honeydew excreted by the insects. The yellow-orange filter papers turned blue when honeydew was absorbed. Before infestation, the insects of both strains were starved for 2 h in a plastic box with a paper towel saturated with distilled water to maintain hydration. Each plant was infested by two large-abdomen brachypterous adult female BPHs. At 24 h, the filter papers were collected and the area of honeydew was measured in ImageJ software (v. 1.53e; NIH, USA; https://imagej.nih.gov/ij).

### Antixenosis test

One plant each of a pre-NIL and Sagabiyori were sown together in a 215-mL plastic cup, with five replicates. At 30 DAS, the plants in each cup were covered with plastic tubes with ventilators. Into each tube, we placed 20 second-instar BPH nymphs. The number of insects that settled on each plant was recorded every day until 5 DAI. The antixenosis level was calculated as the percentage of insects settled on each plant per total in each tube.

### Tolerance test

The tolerance of each line was tested as described by Heinrichs *et al.* (1985). Individual plants of pre-NILs, Sagabiyori, and Rathu Heenati were sown in 1-L plastic cups with three replications. At 45 DAS, each plant was covered with a plastic tube with ventilation, and 100 second- and third-instar BPH nymphs were infested into each tube. Another three cups were covered with a plastic tube were maintained without BPH as controls. When the susceptible control cultivar died, the plants were cut at the soil surface and weighed. The percentage of plant fresh weight loss (PFWL) was used as an inverse measure of tolerance. PFWL is calculated as:

$$\text{PFWL (\%) = }\frac{\text{Fresh weight of control plants – Fresh weight of infested plants}}{\text{Fresh weight of control plants}}\text{×100\%}$$

### Characterization of agronomic traits in NILs

The BC_5_F_2_ Saga-*BPH* NILs and Sagabiyori were grown in a paddy field at Saga University in 2021, and their agronomic traits were characterized. Seedlings were transplanted at 30 DAS at one plant per hill, with 20 cm between hills and 25 cm between rows. Each line was planted in at least three rows (8 plants per row). We measured six agronomic traits (six plants per trait) in the same row: days to heading (DTH), culm length (CL), panicle length (PL), leaf length (LL), leaf width (LW), and panicle number (PN). DTH was assessed as the days from sowing until 50% of panicles flowered. CL was measured from the soil surface to the panicle neck. PL is the length from the panicle neck to the tip of the longest panicle. Flag leaf length (LL) and flag leaf width (LW) were measured on the longest flag leaf of each sampled plant. Panicle number (PN) is the number of reproductive panicles of each plant at maturity.

### Statistical analysis

Mean values of BPH resistance (DS and ADM) of the NILs and agronomic traits were compared by one-way ANOVA. For multiple comparisons we used Tukey–Kramer test for BPH resistance and the Dunnett’s test for agronomic traits in R v. 4.1.2 software.

## Results

### Development of seven NILs for BPH resistance

Seven pre-NILs and seven NILs with BPH resistance genes were developed through MAS and backcrossing (Table 1; Fig. 1). Using 26 markers on **Chr. 4**, we detected the donor *BPH17-ptb* chromosomal segment between RM518 and RM401 (2.0–13.2 Mbp) in Saga-*BPH17-ptb*; *BPH17* between RM518 and RM5749 (2.0–20.1 Mbp) in Saga-*BPH17*; and *BPH20* between RM518 and RM1205 (2.0–19.6 Mbp) in Saga-*BPH20* (Fig. 2A). Using 21 markers on **Chr. 6**, we detected the donor *BPH3* segment between RM6775 and RM588 (0.2–1.6 Mbp) in Saga-*BPH3*; and *BPH32* between RM6775 and RM204 (0.2–3.2 Mbp) in Saga-*BPH32* (Fig. 2B). Using 18 markers on **Chr. 12**, we detected the donor *BPH2* segment between RM28404 and S12091B (21.9–23.7 Mbp) in Saga-*BPH2*, in addition to a long heterozygous region at RM101–RM1246 (8.8–19.2 Mbp); and *BPH21* between RM1986 and S12091B (21.3–23.7 Mbp) in Saga-*BPH21* (Fig. 2C). These results show that the targeted resistance genes from the donor parents were introduced into Sagabiyori.

For surveying genetic background of each NIL, 25575 GRAS-Di markers those were distributed on 12 rice chromosomes were used for genotyping. Based on genotyping by GRAS-Di, Saga-*BPH2* had the substituted chromosomal segments of donor from 19.65 Mbp to 23.96 Mbp on chromosome 12 and from 2.85 Mbp to 5.27 Mbp on chromosome 6. Saga-*BPH17-ptb* had the substituted chromosomal segments of donor from 1.12 Mbp to 14.10 Mbp on chromosome 4. Saga-*BPH32* had the substituted chromosomal segments of donor from 0.13 Mbp to 6.26 Mbp on chromosome 6. Saga-*BPH3* had the substituted chromosomal segments of donor from 0.11 Mbp to 1.65 Mbp on chromosome 6. Saga-*BPH17* had the substituted chromosomal segments of donor from 1.23 Mbp to 20.26 Mbp on chromosome 4. Saga-*BPH20* had the substituted chromosomal segments of donor from 0.14 Mbp to 7.98 Mbp on chromosome 4. Saga-*BPH21* had the substituted chromosomal segments of donor from 19.19 Mbp to 24.55 Mbp on chromosome 12 and from 7.65 Mbp to 20.35 Mbp on chromosome 2 (Supplemental Table 2). Additionally, the genetic background recovery of Sagabiyori was estimated based on genotypes of GRAS-Di markers: 98.2% for Saga-*BPH2*, 96.52% for Saga-*BPH17-ptb*, 98.36% for Saga-*BPH32*, 99.59% for Saga-*BPH3*, 94.9% for Saga-*BPH17*, 97.9% for Saga-*BPH20*, and 95.16% for Saga-*BPH21*, respectively (Supplemental Table 2).

### Screening test with Hadano-1966 and Koshi-2013 populations

**Fig. 3.**
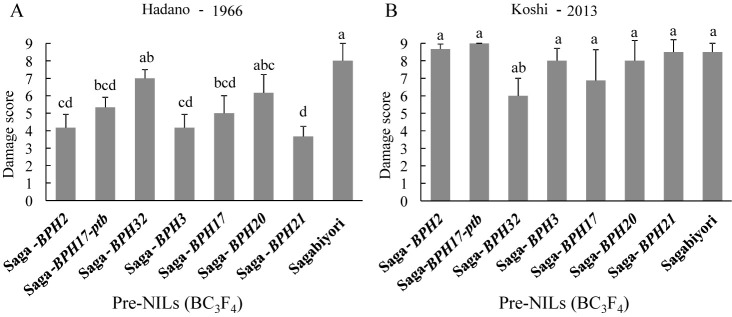
Results of screening test of pre-NILs carrying BPH resistance genes with two BPH populations: Damage score (DS) against A. Hadano-1966 and B. Koshi-2013 populations. Bars with the same letter are not significantly different at *P* < 0.05 by Tukey–Kramer test.

Sagabiyori was highly damaged by both the Hadano-1966 and Koshi-2013 BPH populations (DS ≈ 8; Fig. 3A, 3B, Supplemental Fig. 2). Across pre-NILs, DSs against Hadano-1966 ranged from 3.7 to 7.0. DSs of Saga-*BPH2* (4.2), Saga-*BPH17-ptb* (5.3), Saga-*BPH3* (4.2), Saga-*BPH17* (5.0), and Saga-*BPH21* (3.7) were significantly lower than that of Sagabiyori (8.0; Fig. 3A). Against the more virulent Koshi-2013, DSs of Saga-*BPH32* (6.0) and Saga-*BPH17* (6.9) were marginally lower than that of Sagabiyori (8.5). DSs of the other pre-NILs were almost the same as that of Sagabiyori (Fig. 3B). Thus, most of the pre-NILs were more resistant than Sagabiyori to Hadano-1966, although less resistant to Koshi-2013 than to Hadano-1966.

### Antibiosis test with Hadano-1966 and Koshi-2013 populations

**Fig. 4.**
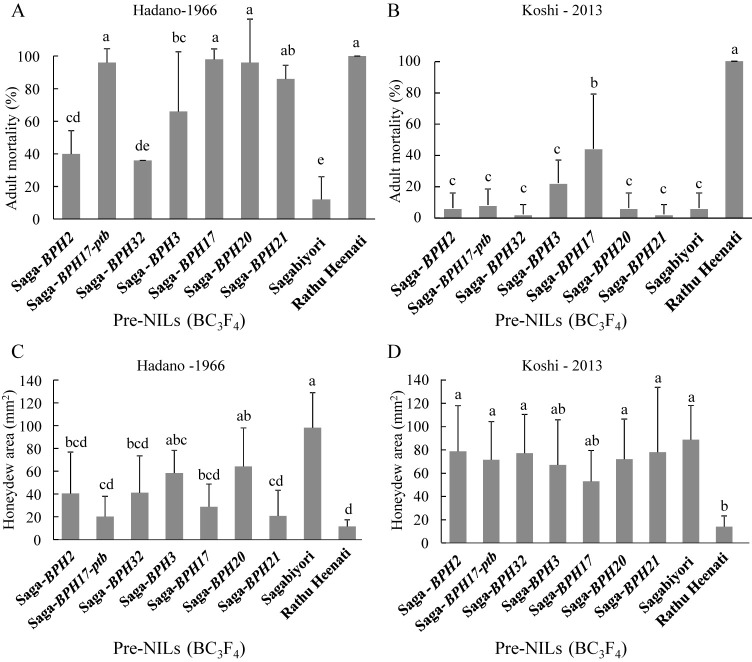
Antibiosis test of pre-NILs carrying BPH resistance genes against two BPH populations. A. and B. Adult mortality against A. Hadano-66 and B. Koshi-2013 populations. C. and D. Honeydew area of C. Hadano-66 and D. Koshi-2013 populations at 24 h after infestation. Bars with the same letter are not significantly different at *P* < 0.05 by Tukey–Kramer test.

At 5 DAI, the ADM was significantly higher on Rathu Heenati (100%) than on Sagabiyori (12% against Hadano-1966, 6% against Koshi-2013; Fig. 4A, 4B). Against Hadano-1966, the ADMs on most of the NILs (Saga-*BPH17-ptb*, 96%; Saga-*BPH3*, 66%; Saga-*BPH17*, 98%; Saga-*BPH20*, 96%; Saga-*BPH21*, 86%) were significantly higher than that on Sagabiyori (Fig. 4A). Against Koshi-2013, however, the ADMs on most of the pre-NILs were the same as that on Sagabiyori, and only that on Saga-*BPH17* (44%) was significantly higher and that on Saga-*BPH3*, 22%, was marginally higher than that on Sagabiyori (6%; Fig. 4B).

Honeydew excretion was measured as an indicator of BPH feeding on the pre-NILs. At 24 h after infestation, the honeydew excretion areas were significantly smaller on Rathu Heenati (11.3 mm^2^ against Hadano-1966, 12.5 mm^2^ against Koshi-2013) than those on Sagabiyori (98.2 and 88.3 mm^2^, respectively; Fig. 4C, 4D). Against Hadano-1966, those on Saga-*BPH17-ptb* (20 mm^2^), Saga-*BPH32* (41 mm^2^), Saga-*BPH17* (28.8 mm^2^), and Saga-*BPH21* (20.6 mm^2^) were significantly smaller than that on Sagabiyori (98.2 mm^2^; Fig. 4C). Against Koshi-2013, those on most of the pre-NILs were same as that on Sagabiyori (88.3 mm^2^), but those on Saga-*BPH3* (66.8 mm^2^) and Saga-*BPH17* (52.4 mm^2^) were marginally smaller (Fig. 4D).

Thus, the Koshi-2013 BPH population had higher virulence than the Hadano-1966 BPH population on the pre-NILs. Nevertheless, Saga-*BPH3* and Saga-*BPH17* showed antibiosis effects against the Koshi-2013 population (Fig. 4B, 4D).

### Antixenosis test with Hadano-1966 and Koshi-2013 populations

**Fig. 5.**
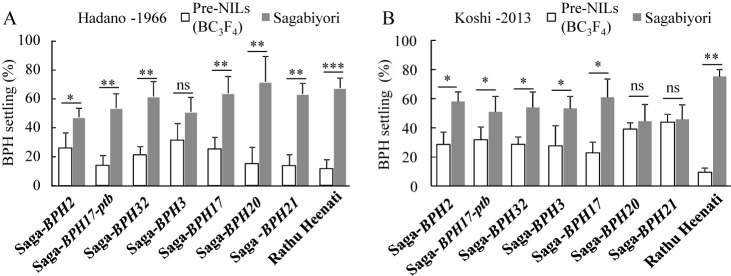
Antixenosis test of pre-NILs carrying BPH resistance genes against two BPH populations. BPH settling against A. Hadano-1966 and B. Koshi-2013 populations. Asterisks indicate significant differences between pre-NILs and Sagabiyori: * *P* < 0.05, ** *P* < 0.01, *** *P* < 0.001 by *t*-test.

We compared the degrees of antixenosis of the seven BPH resistance genes by comparing the numbers of BPH that settled on pairs of each NIL and Sagabiyori at 5 DAI (Fig. 5). The number of BPH was always lower on the NILs than on Sagabiyori (means: Hadano-1966, 20% vs. 57%; Koshi-2013, 31.8% vs. 52.5%). BPH settling behavior differed significantly between Rathu Heenati (11.3% of Hadano-1966; 9.5% of Koshi-2013) and Sagabiyori (66.4% and 75.3%, respectively; Fig. 5A, 5B). Against Hadano-1966, BPH settling percentages were significantly lower on Saga-*BPH2* (25.5%), Saga-*BPH17-ptb* (13.5%), Saga-*BPH32* (20.9%), Saga-*BPH17* (24.9%), Saga-*BPH20* (13.5%), and Saga-*BPH21* (14.7%) than on Sagabiyori (56.8%), and marginally lower on Saga-*BPH3* (31.0%) (Fig. 5A). Against Koshi-2013, BPH settling percentages were significantly lower on Saga-*BPH2* (28.6%), Saga-*BPH17-ptb* (31.8%), Saga-*BPH32* (28.6%), Saga-*BPH3* (27.5%), and Saga-*BPH17* (22.9%) than on Sagabiyori, and slightly lower on Saga-*BPH20* (39.1%) and Saga-*BPH21* (43.8%) (Fig. 5B). Thus, pre-NILs with a single BPH resistance gene could reduce the settling of both populations.

### Tolerance test with Koshi-2013 population

**Fig. 6.**
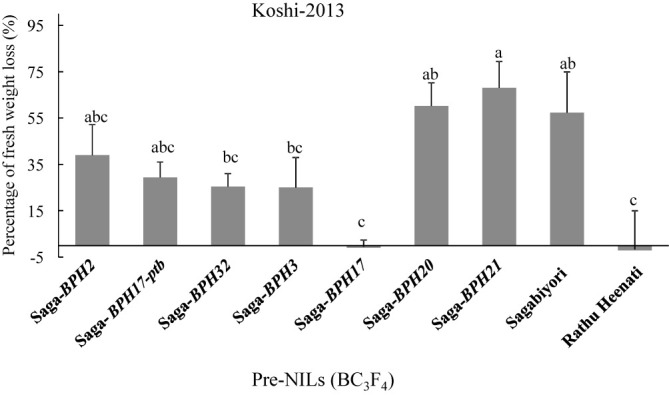
The percent of fresh plant weight loss on Pre-NILs carrying BPH resistance genes against Koshi-2013 BPH population through tolerance test. Bars with the same letter are not significantly different at *P* < 0.05 by Tukey–Kramer test.

Against the Koshi-2013 population, Saga-*BPH20* (60.2%), Saga-*BPH21* (68.0%), and Sagabiyori (57.3%) had the highest PFWL, significantly higher than that of Rathu Heenati (–2.1%; Fig. 6). Saga-*BPH17* (–1.0%) had the lowest PFWL among the pre-NILs and thus the highest tolerance. The PFWLs of Saga-*BPH2* (38.9%), Saga-*BPH17-ptb* (29.4%), Saga-*BPH32* (25.4%), and Saga-*BPH3* (25.1%) were marginally lower than that of Sagabiyori. Thus, most of the pre-NILs had a higher tolerance than Sagabiyori.

### Agronomic characterization of NILs

We evaluated morphological differences between Sagabiyori and the seven NILs in six agronomic traits (Table 2). PL, LW, and PN were not significantly different from those of Sagabiyori. DTH for NILs were similar to that of Sagabiyori, except that those of Saga-*BPH17* and Saga-*BPH20* were slightly and significantly shorter. CLs and LLs of most of the NILs were not significantly different from those of Sagabiyori, but CLs of Saga-*BPH2* and Saga*-BPH21* were longer and that of Saga-*BPH17* was shorter, and LLs of Saga-*BPH3* and Saga-*BPH20* were shorter and that of Saga-*BPH-17ptb* was longer. Overall, the agronomic characteristics of most of the NILs were similar to those of Sagabiyori.

## Discussion

Outbreaks of BPH in western and southwestern Japan in 2013 and 2019 cost Japanese rice farmers greatly (Fujii *et al.* 2021). As the commercial varieties grown in these regions do not have BPH resistance genes, it is essential to introduce BPH resistance into them. Since the early 1970s, resistant varieties have been used extensively in breeding for BPH resistance in *japonica* varieties, such as ‘Norin-PL4’ (Murata *et al.* 1998), ‘Saikai 190’ (Kobayashi *et al.* 2014), ‘Kanto *BPH1*’, and ‘Akiharuka’. However, these varieties are not widely grown in Japan. Therefore, the improvement of the BPH resistance of the commercial variety Sagabiyori is necessary for future rice production. The seven NILs for BPH resistance in the Sagabiyori background that we bred can be used as commercial cultivars or breeding materials.

In general, NILs are used for genetic studies such as the characterization of gene effects, development of molecular markers linked with target genes, comparisons of gene expression, and isolation of target genes (Fujita *et al.* 2010, Xiao *et al.* 2016). In the context of BPH, they are important for monitoring virulence among differential BPH populations in Japan (Myint *et al.* 2009b, Nguyen *et al.* 2019), East Asia (Fujita *et al.* 2009), and the Philippines (Jena *et al.* 2017). By monitoring BPH virulence using our NILs, we can determine the effectiveness of resistance genes against specific BPH populations. Here, almost all of the NILs were resistant to Hadano-1966 but less effective against Koshi-2013 (Figs. 3–6). The virulence of Koshi-2013 BPH was significantly greater than that of Hadano-1966, and the single genes in the pre-NILs were less effective (Figs. 3, 4). These tendencies of BPH virulence correspond with studies monitoring the virulence of BPH populations in Japan (Fujii *et al.* 2021, Nguyen *et al.* 2019).

The virulence of BPH on differential cultivars fluctuates by location and year. BPH has adapted to resistant cultivars containing *BPH1*, *BPH2*, *BPH3*, *BPH5*, *BPH7*, *BPH8*, *BPH9*, *BPH10*, and *BPH18* across Asia. In Japan, resistance in differential cultivars with *BPH1* and *BPH2* broke down during the 1990s (Tanaka and Matsumura 2000). The virulence of BPH populations collected in 1999 (Isahaya-99), 2005 (Nishigoshi-05), and 2013 (Koshi-2013) was significantly greater than that of Hatano-1966 (Myint *et al.* 2009b, Nguyen *et al.* 2019). Here, most of the NILs were resistant to Hadano-1966 but weak against Koshi-2013 (Figs. 3–5). These results suggest that BPH virulence has increased year by year, and that *BPH2*, *BPH17-ptb*, *BPH32*, *BPH20*, and *BPH21* are now less effective against Koshi-2013. The BPH settling test showed that the pre-NILs carrying single resistance genes are more resistant than Sagabiyori to Koshi-2013, although their antibiosis effects were less effective against Koshi-2013 (Fig. 5). Although all of the pre-NILs were weak against Koshi-2013, most of them deterred it.

Among the seven NILs, Saga-*BPH3* and Saga-*BPH17* (from Rathu Heenati) were resistant to the migrant Koshi-2013 population. The resistance of Saga-*BPH17* to Koshi-2013 was significantly higher than that of Sagabiyori in antibiosis, antixenosis, and tolerance tests (Figs. 4–6). The resistance of Saga-*BPH3* was marginally higher than that of Sagabiyori in antibiosis and tolerance tests (Figs. 4, 6). *BPH3* and *BPH17* in Rathu Heenati were reported as offering a broad spectrum of resistance to various BPH populations in Asia (Fujii *et al.* 2021, Horgan *et al.* 2015, Myint *et al.* 2009b). The *BPH3* gene in Rathu Heenati, mapped on the short arm of Chr. 6 between markers RM19291 (1.2 Mbp) and RM8072 (1.4 Mbp), conferred strong resistance to Thai BPH populations (Jairin *et al.* 2007a, 2007b). It also conferred strong resistance in the genetic background of *indica* cultivars ‘Hemeizhan’ and ‘9311’ to field populations in China (Hu *et al.* 2016b, Xiao *et al.* 2016). Our results agree with those studies. In contrast, the resistance of *BPH3* to Koshi-2013 was lower in the T65 genetic background (Nguyen *et al.* 2019). Therefore, the resistance effect of *BPH3* might depend on the genetic background or the BPH population. *BPH17* encodes plasma membrane–localized lectin receptor kinases *OsLecRK1*–*3*; (Liu *et al.* 2015), while most BPH resistance genes, such as *BPH14*, *BPH18*, and *BPH26*, encode coiled-coil nucleotide-binding-site leucine-rich repeats (CC-NBS-LRR). The functions of three genes in a cluster of four at *BPH17* contribute to broad-spectrum durable BPH resistance effects (Liu *et al.* 2015). This could explain the stronger effects of *BPH17* against Koshi-2013.

Resistant cultivars carrying multiple BPH resistance genes had greater or more durable resistance (Jena *et al.* 2017, Myint *et al.* 2009a, Nguyen *et al.* 2019, Xiao *et al.* 2016). For example, PYLs *BPH6* + *BPH12* (Qiu *et al.* 2012), *BPH3* + *BPH27* (Liu *et al.* 2016), *BPH25* + *BPH26* (Myint *et al.* 2012), and *BPH17* + *BPH21* (Jena *et al.* 2017) had higher resistance than monogenic lines with each of these genes alone. Here, the resistance of Saga-*BPH3* and Saga-*BPH17* was higher than that of Sagabiyori (Figs. 4–6). From the DS and ADM results, the resistance of *BPH17* was stronger than that of *BPH3*, indicating that *BPH17* would be a useful gene resource for improving BPH resistance in rice breeding programs. Through pyramiding *BPH3* and *BPH17*, lines carrying both resistance genes might have stronger antibiosis, antixenosis, and tolerance according to resistance level of Saga-*BPH3* and Saga-*BPH17* in this study. In Nguyen *et al.* (2019), PYL carrying *BPH3* and *BPH17* with T65 genetic background had been developed and characterized ADM against Koshi-2013. The ADM of PYL carrying *BPH3* and *BPH17* with T65 genetic background against Koshi-2013 was higher than that of corresponding NILs but was lower than that of Rathu Heenati. The difference in ADM between PYL and Rathu Heenati might be due to BPH resistance genes in Rathu Heenati except *BPH3* and *BPH17* such as *BPH14* (Pannak *et al.* 2023), *Qbph3*, and *Qbph10* (Sun *et al.* 2005).

Moreover, our new NILs will facilitate the development of PYLs carrying *BPH3* and *BPH17*, which will be important for increasing resistance to the Koshi-2013 population. Although most of the NILs developed here have less resistance to Koshi-2013, pyramiding of BPH resistance genes might enhance resistance. In future research, it will be essential to develop PYLs as possible resources for breeding programs. Recently, the development of heat tolerance rice varieties become important because of climate changes. The rice varieties with heat tolerance such as ‘Nikomaru’ and ‘Genkitsukushi’ has been developed in Japan and there was tendency that heat tolerance rice varieties have high NSC content (Tanamachi *et al.* 2016). Current study revealed that the Sagabiyori background with high NSC content became stronger resistance by transferring *BPH3* and *BPH17* genes. These BPH resistance genes would be effective on the other rice varieties with high NSC content.

## Author Contribution Statement

SBDS, SZ, HY, and DF designed the study. SBDS, CDN, and DF developed the plant materials. SSM provided BPH populations. SBDS and DF performed the experiments and wrote the paper.

## Supplementary Material

Supplemental Figures

Supplemental Tables

## Figures and Tables

**Table 1.** Development of pre-NILs and NILs for BPH resistance genes in Sagabiyori genetic background

Line	Gene	Chromosome	Donor	Pre-NIL generation	NIL generation
Saga-*BPH2*	*BPH2*	12	PTB33	BC_3_F_4_	BC_5_F_2_
Saga-*BPH17-ptb*	*BPH17-ptb*	4	PTB33	BC_3_F_4_	BC_5_F_2_
Saga-*BPH32*	*BPH32*	6	PTB33	BC_3_F_4_	BC_5_F_2_
Saga-*BPH3*	*BPH3*	6	Rathu Heenati	BC_3_F_4_	BC_5_F_2_
Saga-*BPH17*	*BPH17*	4	Rathu Heenati	BC_3_F_4_	BC_5_F_2_
Saga-*BPH20*	*BPH20*	4	IR71033-121-15	BC_3_F_4_	BC_5_F_2_
Saga-*BPH21*	*BPH21*	12	IR71033-121-15	BC_3_F_4_	BC_5_F_2_

**Table 2.** Agronomic traits of NILs (BC_5_F_2_) for brown planthopper resistance genes

Line	Agronomic trait (mean ± SD)					
	DTH	CL (cm)	PL (cm)	LL (cm)	LW (cm)	PN
Saga-*BPH2*	75.0 ± 0.5	90.8 ± 4.7**	17.5 ± 0.5	33.6 ± 6.6	1.2 ± 0.0	15.7 ± 2.1
Saga-*BPH17-ptb*	74.5 ± 1.1	83.6 ± 4.9	18.6 ± 1.0	36.2 ± 3.2*	1.3 ± 0.1	16.6 ± 3.5
Saga-*BPH32*	74.0 ± 1.0	83.3 ± 2.4	18.3 ± 0.9	34.0 ± 2.8	1.3 ± 0.0	13.5 ± 1.5
Saga-*BPH3*	74.0 ± 1.2	82.9 ± 3.3	17.8 ± 0.9	23.4 ± 2.4*	1.3 ± 0.1	17.7 ± 3.9
Saga-*BPH17*	72.0 ± 0.8*	72.8 ± 3.7**	19.0 ± 1.1	26.2 ± 5.3	1.3 ± 0.1	15.1 ± 2.5
Saga-*BPH20*	67.0 ± 0.5*	80.5 ± 2.4	18.0 ± 1.3	22.6 ± 1.9**	1.3 ± 0.1	11.8 ± 2.4
Saga-*BPH21*	76.0 ± 1.3	89.9 ± 4.4**	19.7 ± 0.4	27.3 ± 4.1	1.3 ± 0.1	14.4 ± 3.8
Sagabiyori	75.3 ± 1.2	79.2 ± 3.3	19.0 ± 1.2	30.6 ± 3.9	1.3 ± 0.1	15.5 ± 2.9

DTH, days to heading; CL, culm length; PL, panicle length; LL, flag leaf length; LW, flag leaf width; PN, panicle number per plant. * *P* < 0.05, ** *P* < 0.01 (Dunnett’s test with Sagabiyori as the control).

## References

[B1] Angeles, E.R., G.S. Khush and E.A. Heinrichs (1986) Inheritance of resistance to planthoppers and leafhoppers in rice. *In*: Rice Genetics, Proceedings of the International Rice Genetics Symposium, International Rice Research Institute, Manila, pp. 537–549.

[B2] Bottrell, D.G. and K.G. Schoenly (2012) Resurrecting the ghost of green revolutions past: The brown planthopper as a recurring threat to high-yielding rice production in tropical Asia. J Asia Pac Entomol 15: 122–140.

[B3] Brar, D.S., P.S. Virk, K.K. Jena and G.S. Khush (2009) Breeding for resistance to planthoppers in rice. *In*: Heong, K.L. and B. Hardy (eds.) Planthopper: new threats to the sustainability of intensive rice production systems in Asia, International Rice Research Institute, Los Baños, pp. 401–427.

[B4] Claridge, M.F. and J. Den Hollander (1982) Virulence to rice cultivars and selection for virulence in populations of the brown planthopper *Nilaparvata lugens*. Entomol Exp Appl 32: 213–221.

[B5] Dellaporta, S.L., J. Wood and J.B. Hicks (1983) A plant DNA minipreparation: Version II. Plant Mol Biol Report 1: 19–21.

[B6] Du, B., W. Zhang, B. Liu, J. Hu, Z. Wei, Z. Shi, R. He, L. Zhu, R. Chen, B. Han et al. (2009) Identification and characterization of *Bph14*, a gene conferring resistance to brown planthopper in rice. Proc Natl Acad Sci USA 106: 22163–22168. 20018701 10.1073/pnas.0912139106 PMC2793316

[B7] Du, B., R. Chen, J. Guo and G. He (2020) Current understanding of the genomic, genetic, and molecular control of insect resistance in rice. Mol Breed 40: 24.

[B8] Dyck, V.A. and B. Thomas (1979) The brown planthopper problem. *In*: Brown planthopper: threat to rice production in Asia, International Rice Research Institute, Los Baños, pp. 3–13.

[B9] Ferrater, J.B., A.I. Naredo, M.L.P. Almazan, P.W. de Jong, M. Dicke and F.G. Horgan (2015) Varied responses by yeast-like symbionts during virulence adaptation in a monophagous phloem-feeding insect. Arthropod Plant Interact 9: 215–224.

[B10] Fujii, T., K. Yoshida, T. Kobayashi, K.K.M. Myint, H. Yasui, S. Sanada-Morimura and M. Matsumura (2021) Long-term virulence monitoring of differential cultivars in Japan’s immigrant populations of *Nilaparvata lugens* (Hemiptera: Delphacidae) in 2001–2019. Appl Entomol Zool 56: 407–418.

[B11] Fujita, D., K.K.M. Myint, M. Matsumura and H. Yasui (2009) The genetics of host plant resistance to rice planthopper and leafhopper. *In*: Heong, K.L. and B. Hardy (eds.) Planthoppers: New threats to the sustainability of intensive rice production systems in Asia, International Rice Research Institute, Los Baños, pp. 389–399.

[B12] Fujita, D., A. Yoshimura and H. Yasui (2010) Development of near-isogenic lines and pyramided lines carrying resistance genes to green rice leafhopper (*Nephotettix cincticeps* Uhler) with the Taichung 65 genetic background in rice (*Oryza sativa* L.). Breed Sci 60: 18–27.

[B13] Fujita, D., A. Kohli and F.G. Horgan (2013) Rice resistance to planthoppers and leafhoppers. CRC Crit Rev Plant Sci 32: 162–191.

[B14] Guo, J., C. Xu, D. Wu, Y. Zhao, Y. Qiu, X. Wang, Y. Ouyang, B. Cai, X. Liu, S. Jing et al. (2018) *Bph6* encodes an exocyst-localized protein and confers broad resistance to planthoppers in rice. Nat Genet 50: 297–306. 29358653 10.1038/s41588-018-0039-6

[B15] Heinrichs, E.A., F.G. Medrano and H.R. Rapusas (1985) Brown plant-hopper, whitebacked planthopper, green leafhopper, and zigzag leafhopper. *In*: Heinrichs, E.A., F.G. Medrano and H.R. Rapusas (eds.) Genetic evaluation for insect resistance in rice, International Rice Research Institute, Los Baños, pp. 71–170.

[B16] Horgan, F.G., A.F. Ramal, J.S. Bentur, R. Kumar, K.V. Bhanu, P.S. Sarao, E.H. Iswanto, H.V. Chien, M.H. Phyu, C.C. Bernal et al. (2015) Virulence of brown planthopper (*Nilaparvata lugens*) populations from South and South East Asia against resistant rice varieties. Crop Prot 78: 222–231.

[B17] Hosoya, S., S. Hirase, K. Kikuchi, K. Nanjo, Y. Nakamura, H. Kohno and M. Sano (2019) Random PCR-based genotyping by sequencing technology GRAS-Di (genotyping by random amplicon sequencing, direct) reveals genetic structure of mangrove fishes. Mol Ecol Resour 19: 1153–1163. 31009151 10.1111/1755-0998.13025

[B18] Hu, G., M.-H. Lu, H.A. Tuan, W.-C. Liu, M.-C. Xie, C.E. McInerney and B.-P. Zhai (2017) Population dynamics of rice planthoppers, *Nilaparvata lugens* and *Sogatella furcifera* (Hemiptera, Delphacidae) in Central Vietnam and its effects on their spring migration to China. Bull Entomol Res 107: 369–381. 27919313 10.1017/S0007485316001024

[B19] Hu, J., C. Xiao and Y.Q. He (2016a) Recent progress on the genetics and molecular breeding of brown planthopper resistance in rice. Rice (N Y) 9: 30. 27300326 10.1186/s12284-016-0099-0 PMC4908088

[B20] Hu, W., H. Xiao, K. Hu, Y.J. Jiang and Y. Zhang (2016b) Application of marker-assisted backcross to introgress *Bph3*, *Bph14* and *Bph15* into an elite *indica* rice variety for improving its resistance to brown planthopper. Plant Breed 135: 291–300.

[B21] International Rice Research Institute (IRRI) (2014) Standard evaluation system for rice (SES) 5th ed. International Rice Research Institute, Los Baños, p. 57.

[B22] Jairin, J., K. Phengrat, S. Teangdeerith, A. Vanavichit and T. Toojinda (2007a) Mapping of a broad-spectrum brown planthopper resistance gene, *Bph3*, on rice chromosome 6. Mol Breed 19: 35–44.

[B23] Jairin, J., S. Teangdeerith, P. Leelagud, K. Phengrat, A. Vanavichit and T. Toojinda (2007b) Physical mapping of *Bph3*, a brown plant-hopper resistance locus in rice. Maejo International Journal of Science and Technology 1: 166–177.

[B24] Jena, K.K., S.L. Hechanova, H. Verdeprado, G.D. Prahalada and S.R. Kim (2017) Development of 25 near-isogenic lines (NILs) with ten BPH resistance genes in rice (*Oryza sativa* L.): production, resistance spectrum, and molecular analysis. Theor Appl Genet 130: 2345–2360. 28795219 10.1007/s00122-017-2963-8

[B25] Ji, H., S.R. Kim, Y.H. Kim, J.P. Suh, H.M. Park, N. Sreenivasulu, G. Misra, S.M. Kim, S.L. Hechanova, H. Kim et al. (2016) Map-based cloning and characterization of the *BPH18* gene from wild rice conferring resistance to brown planthopper (BPH) insect pest. Sci Rep 6: 34376. 27682162 10.1038/srep34376 PMC5041133

[B26] Ketipearachchi, Y., C. Kaneda and C. Nakamura (1998) Adaptation of the brown planthopper (BPH), *Nilaparvata lugens* (Stål) (Homoptera: Delphacidae), to BPH resistant rice cultivars carrying *bph8* or *Bph9*. Appl Entomol Zool 33: 497–505.

[B27] Khush, G.S. (1995) Modern varieties—Their real contribution to food supply and equity. GeoJournal 35: 275–284.

[B28] Kobayashi, T., K. Yamamoto, Y. Suetsugu, S. Kuwazaki, M. Hattori, J. Jairin, S. Sanada-Morimura and M. Matsumura (2014) Genetic mapping of the rice resistance-breaking gene of the brown planthopper *Nilaparvata lugens*. Proc R Soc Lond B Biol Sci 281: 20140726. 10.1098/rspb.2014.0726 PMC4071553 24870048

[B29] Liu, Y.L., L.M. Chen, Y.Q. Liu, H.M. Dai, J. He, H.Y. Kang, G. Pan, J. Huang, Z.Y. Qiu, Q. Wang et al. (2016) Marker assisted pyramiding of two brown planthopper resistance genes, *Bph3* and *Bph27*(t), into elite rice cultivars. Rice (N Y) 9: 27. 27246014 10.1186/s12284-016-0096-3 PMC4887400

[B30] Liu, Y.Q., H. Wu, H. Chen, Y.L. Liu, J. He, H.Y. Kang, Z.G. Sun, G. Pan, Q. Wang, J. Hu et al. (2015) A gene cluster encoding lectin receptor kinases confers broad-spectrum and durable insect resistance in rice. Nat Biotechnol 33: 301–305. 25485617 10.1038/nbt.3069

[B31] Murata, K., M. Fujiwara, C. Kaneda, S. Takumi, N. Mori and C. Nakamura (1998) RFLP mapping of a brown planthopper (*Nilaparvata lugens* Stål) resistance gene *bph2* of *indica* rice introgressed into a *japonica* breeding line ‘Norin-PL4’. Genes Genet Syst 73: 359–364.

[B32] Myint, K.K.M., M. Matsumura, M. Takagi and H. Yasui (2009a) Demographic parameters of long-term laboratory strains of the brown planthopper, *Nilaparvata lugens* Stål, (Homoptera: Delphacidae) on resistance genes, *bph20*(t) and *Bph21*(t) in rice. Journal of the Faculty of Agriculture, Kyushu University 54: 159–164.

[B33] Myint, K.K.M., H. Yasui, M. Takagi and M. Matsumura (2009b) Virulence of long-term laboratory populations of the brown planthopper, *Nilaparvata lugens* (StåI), and whitebacked planthopper, *Sogatella furcifera* (Horváth) (Homoptera: Delphacidae), on rice differential varieties. Appl Entomol Zool 44: 149–153.

[B34] Myint, K.K.M., D. Fujita, M. Matsumura, T. Sonoda, A. Yoshimura and H. Yasui (2012) Mapping and pyramiding of two major genes for resistance to the brown planthopper (*Nilaparvata lugens* [Stål]) in the rice cultivar ADR52. Theor Appl Genet 124: 495–504. 22048639 10.1007/s00122-011-1723-4 PMC3265730

[B35] Nguyen, C.D., H. Verdeprado, D. Zita, S. Sanada-Morimura, M. Matsumura, P.S. Virk, D.S. Brar, F.G. Horgan, H. Yasui and D. Fujita (2019) The development and characterization of near-isogenic and pyramided lines carrying resistance genes to brown planthopper with the genetic background of *Japonica* rice (*Oryza sativa* L.). Plants 8: 498. 31726710 10.3390/plants8110498 PMC6918374

[B36] Nguyen, C.D., S.H. Zheng, S. Sanada-Morimura, M. Matsumura, H. Yasui and D. Fujita (2021) Substitution mapping and characterization of brown planthopper resistance genes from *indica* rice variety, ‘PTB33’ (*Oryza sativa* L.). Breed Sci 71: 497–509. 35087314 10.1270/jsbbs.21034 PMC8784355

[B37] Pannak, S., S. Wanchana, W. Aesomnuk, M.K. Pitaloka, W. Jamboonsri, M. Siangliw, B.C. Meyers, T. Toojinda and S. Arikit (2023) Functional *Bph14* from Rathu Heenati promotes resistance to BPH at the early seedling stage of rice (*Oryza sativa* L.) as revealed by QTL-seq. Theor Appl Genet 136: 25. 36781491 10.1007/s00122-023-04318-w

[B38] Qiu, Y., J. Guo, S. Jing, L. Zhu and G. He (2012) Development and characterization of *japonica* rice lines carrying the brown planthopper-resistance genes *BPH12* and *BPH6*. Theor Appl Genet 124: 485–494. 22038433 10.1007/s00122-011-1722-5

[B39] Rahman, M.L., W. Jiang, S.H. Chu, Y. Qiao, T.H. Ham, M.O. Woo, J. Lee, M.S. Khanam, J.H. Chin, J.U. Jeung et al. (2009) High-resolution mapping of two rice brown planthopper resistance genes, *Bph20(t)* and *Bph21(t)*, originating from *Oryza minuta*. Theor Appl Genet 119: 1237–1246. 19669727 10.1007/s00122-009-1125-z

[B40] Ren, J., F. Gao, X. Wu, X. Lu, L. Zeng, J. Lv, X. Su, H. Luo and G. Ren (2016) *Bph32*, a novel gene encoding an unknown SCR domain-containing protein, confers resistance against the brown planthopper in rice. Sci Rep 6: 37645. 27876888 10.1038/srep37645 PMC5120289

[B41] Sanada-Morimura, S. (2020) Main factors affecting outbreak of the brown planthopper in 2019 in Japan. Plant Protection 74: 231–235 (in Japanese with English summary).

[B42] Saxena, R.C. and A.A. Barrion (1985) Biotypes of the brown planthopper *Nilaparvata lugens* (Stål) and strategies in deployment of host plant resistance. Int J Trop Insect Sci 6: 271–289.

[B43] Shi, S., H. Wang, L. Nie, D. Tan, C. Zhou, Q. Zhang, Y. Li, B. Du, J. Guo, J. Huang et al. (2021) *Bph30* confers resistance to brown planthopper by fortifying sclerenchyma in rice leaf sheaths. Mol Plant 14: 1714–1732. 34246801 10.1016/j.molp.2021.07.004

[B44] Sun, L.H., C.C. Su, C.M. Wang, H.Q. Zhai and J.M. Wan (2005) Mapping of a major resistance gene to the brown planthopper in the rice cultivar Rathu Heenati. Breed Sci 55: 391–396.

[B45] Tamura, Y., M. Hattori, H. Yoshioka, M. Yoshioka, A. Takahashi, J. Wu, N. Sentoku and H. Yasui (2014) Map-based cloning and characterization of a brown planthopper resistance gene *BPH26* from *Oryza sativa* L. ssp. *indica* cultivar ADR52. Sci Rep 4: 5872. 25076167 10.1038/srep05872 PMC5376202

[B46] Tanaka, K. and M. Matsumura (2000) Development of virulence to resistant rice varieties in the brown planthopper, *Nilaparvata lugens* (Homoptera: Delphacidae), immigrating into Japan. Appl Entomol Zool 35: 529–533.

[B47] Tanamachi, K., M. Miyazaki, K. Matsuo, C. Suriyasak, A. Tamada, K. Matsuyama, M. Iwaya-Inoue and Y. Ishibashi (2016) Differential responses to high temperature during maturation in heat-stress-tolerant cultivars of *Japonica* rice. Plant Proc Sci 19: 300–308.

[B48] Van Berloo, R. (2008) GGT 2.0: Versatile software for visualization and analysis of genetic Data. J Hered 99: 232–236. 18222930 10.1093/jhered/esm109

[B49] Wang, X., Y. Han, Y. Zhang, B. Deng, B. Wu, X. Guo, Y. Qin, Y. Fang, F. Liu, B. Qin et al. (2022) QTL mapping integrated with BSA-Seq analysis identifies a novel gene conferring resistance to brown planthopper from common wild rice (*Oryza rufipogon* Griff.). Euphytica 218: 34.

[B50] Wang, Y., L. Cao, Y. Zhang, C. Cao, F. Liu, F. Huang, Y. Qiu, R. Li and X. Luo (2015) Map-based cloning and characterization of *BPH29*, a B3 domain-containing recessive gene conferring brown planthopper resistance in rice. J Exp Bot 66: 6035–6045. 26136269 10.1093/jxb/erv318 PMC4566989

[B51] Watanabe, T. and H. Kitagawa (2000) Photosynthesis and translocation of assimilates in rice plants following phloem feeding by the planthopper *Nilaparvata lugens* (Homoptera: Delphacidae). J Econ Entomol 93: 1192–1198. 10985030 10.1603/0022-0493-93.4.1192

[B52] Xiao, C., J. Hu, Y.T. Ao, M.X. Cheng, G.J. Gao, Q.L. Zhang, G.C. He and Y.Q. He (2016) Development and evaluation of near-isogenic lines for brown planthopper resistance in rice cv. 9311. Sci Rep 6: 38159. 27901104 10.1038/srep38159 PMC5128867

[B53] Yang, M., L. Cheng, L. Yan, W. Shu, X. Wang and Y. Qiu (2019) Mapping and characterization of a quantitative trait locus resistance to the brown planthopper in the rice variety IR64. Hereditas 156: 22. 31297040 10.1186/s41065-019-0098-4 PMC6595561

[B54] Young, N.D. and S.D. Tanksley (1989) Restriction fragment length polymorphism maps and the concept of graphical genotypes. Theor Appl Genet 77: 95–101. 24232480 10.1007/BF00292322

[B55] Zhang, Y., G. Qin, Q. Ma, M. Wei, X. Yang, Z. Ma, H. Liang, C. Liu, Z. Li, F. Liu et al. (2020) Identification of major locus *Bph35* resistance to brown planthopper in rice. Rice Sci 27: 237–245.

[B56] Zhao, Y., J. Huang, Z. Wang, S. Jing, Y. Wang, Y. Ouyang, B. Cai, X.F. Xin, X. Liu, C. Zhang et al. (2016) Allelic diversity in an NLR gene *BPH9* enables rice to combat planthopper variation. Proc Natl Acad Sci USA 113: 12850–12855. 27791169 10.1073/pnas.1614862113 PMC5111712

