# Supplementary material for: Development and characterization of near-isogenic lines for brown planthopper resistance genes in the genetic background of japonica rice ‘Sagabiyori’
Source: Breed Sci. 2023 Sep 9;73(4):382–92. doi: 10.1270/jsbbs.23017 (PMC10722098; doi:10.1270/jsbbs.23017)
Supplement: Supplementary file 1 — Supplemental Figures [file 73_382_s1.pdf]

doi: 10.1270/jsbbs.23017

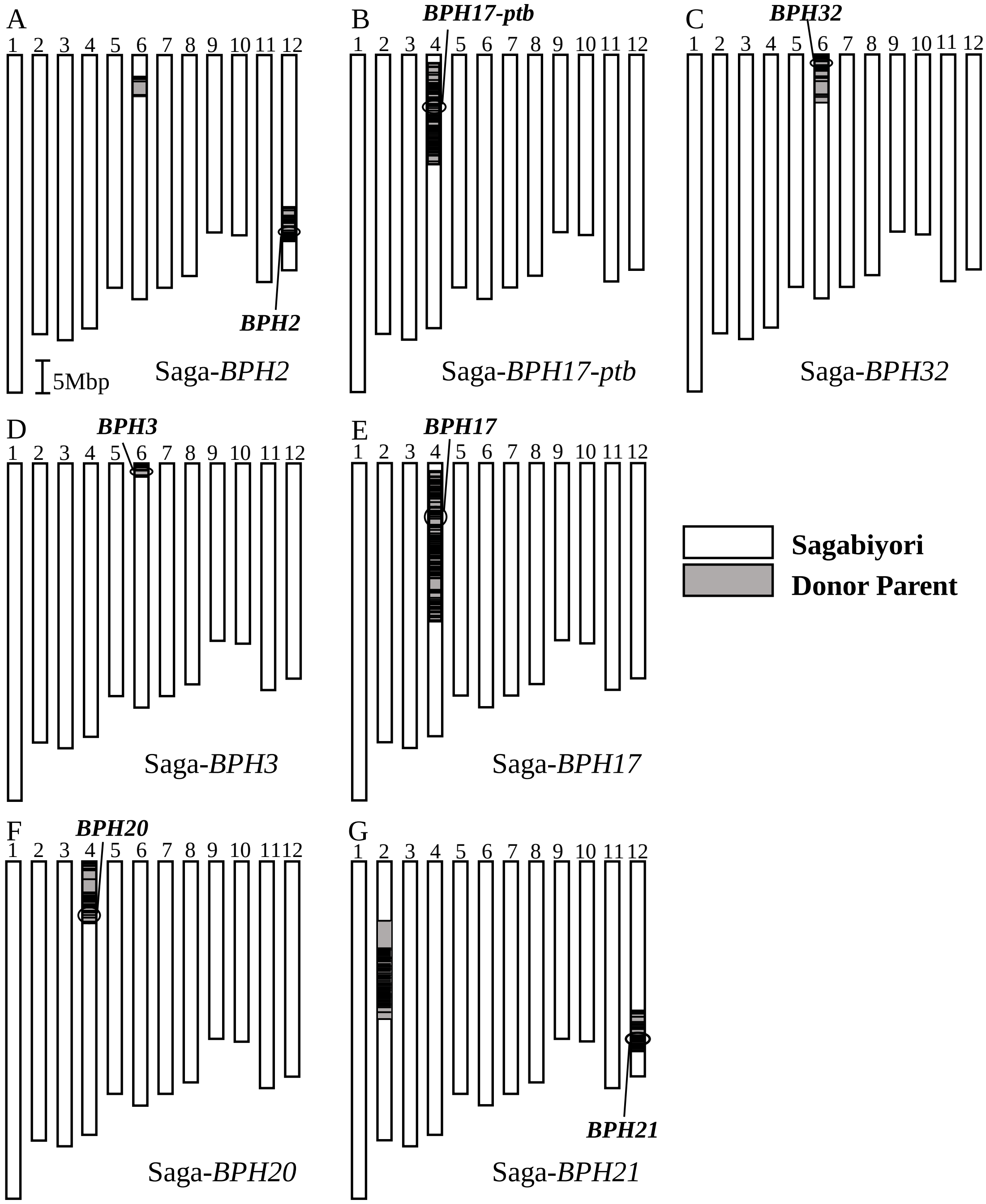

Supplemental Fig. 1 Graphical genotype of *Saga-BPH2* (A), Saga-*BPH17-ptb* (B), Saga-*BPH32* (C), Saga-*BPH3* (D), Saga-*BPH17* (E), Saga-*BPH20* (F), Saga-*BPH21* (G). The 12 bars indicate 12 chromosomes of rice. Horizontal lines across the chromosomes show the positions of polymorphic GRAS-Di markers. Circles indicate the approximate positions of brown planthopper resistant genes.

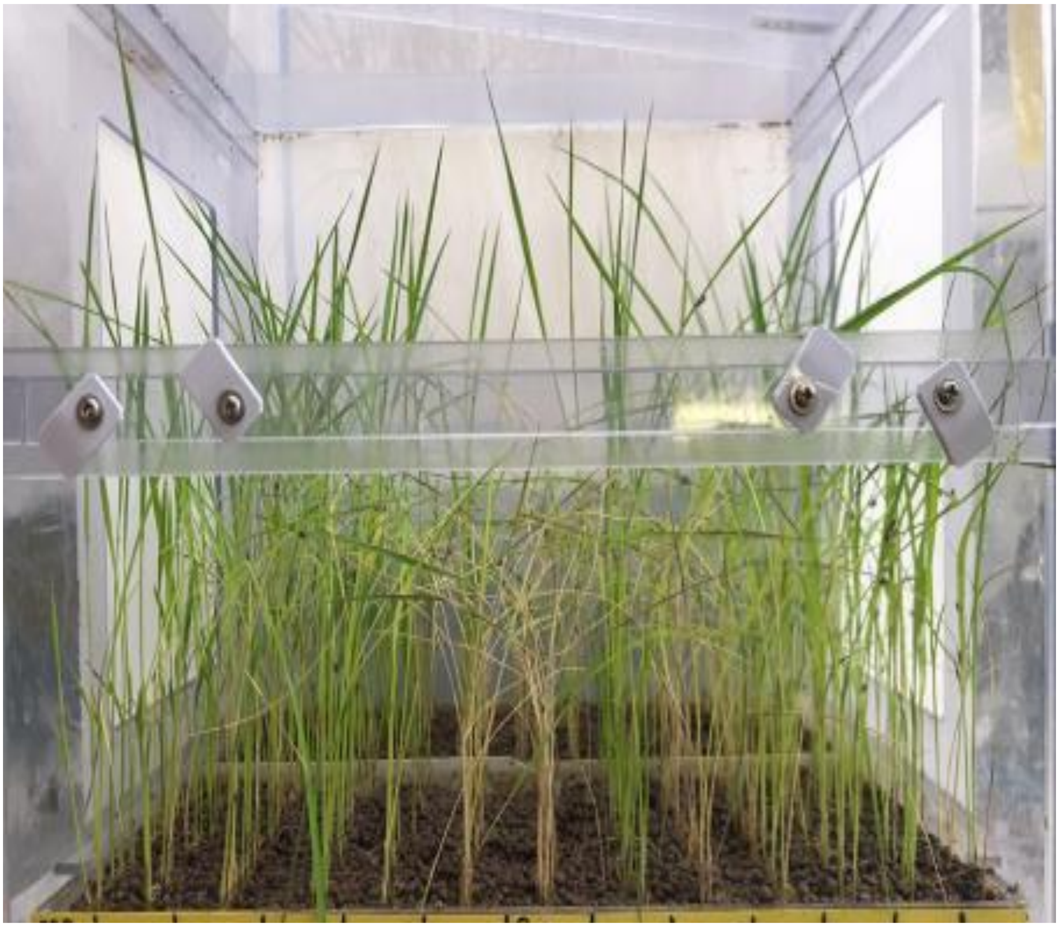

Saga-*BPH3*
Saga-*BPH17-ptb*
Saga-*BPH17*
Saga-*BPH32*
Saga-*BPH2*
Saga-*BPH20*
Sagabiyori
Saga-*BPH21*
Saga-*BPH32*
Saga-*BPH17-ptb*
Saga-*BPH21*
Saga-*BPH2*
Saga-*BPH3*

Supplemental Fig. 2 Modified seed box screening test (MSST) of pre-NILs (BC_3F_4) against Hadano-1966 BPH population
