# Supplementary material for: Development and characterization of near-isogenic lines for brown planthopper resistance genes in the genetic background of japonica rice ‘Sagabiyori’
Source: Breed Sci. 2023 Sep 9;73(4):382–92. doi: 10.1270/jsbbs.23017 (PMC10722098; doi:10.1270/jsbbs.23017)
Supplement: Supplementary file 2 — Supplemental Tables [file 73_382_s2.pdf]

doi: 10.1270/jsbbs.23017

Supplemental Table 1. The SSR markers used for marker-assisted selection of seven genes for resistance to brown planthopper

Markers	Chr.	Resistance genes	Forward primer sequence (5'-3')	Reverse primer sequence (5'-3')	Physical position (Mbp)
RM28404	12	*BPH2*, *BPH21*	GTGGGAGTCGAGAGGC GATAAGG	AAAGGACGGCTCATAG GTGATGG	21.88
RM28493	12	*BPH2*, *BPH21*	ACCGTTAGATGACACA AGCAACG	GGTTAGCAAGACTGGA GGAGACG	23.31
RM1305	4	*BPH17-ptb*, *BPH17*	GGTACTACAAAGAAAC TGCATCG	TCCTAGCTCAAATGTGC TATCTGG	5.62
B40	4	*BPH17-ptb*, *BPH17*	CAATACCGGATATCTTG ACTCC	CGACCACGCTGCCTATA TTC	8.20
RM508	6	*BPH3*, *BPH32*	AGAAGCCGGTTCATAGT TCATGC	ACCCGTGAACCACAAA GAACG	4.42
RM588	6	*BPH3*, *BPH32*	TCTTGCTGTGCTGTTAG TGTACG	GCAGGACATAAATACT AGGCATGG	1.61
RM1305	4	*BPH20*	GGTACTACAAAGAAAC TGCATCG	TCCTAGCTCAAATGTGC TATCTGG	5.62
RM16531	4	*BPH20*	CAGTGCAGGAACAAGA TTCAGG	CATTGCAGTTGGGTTCT ATTGG	7.93

Supplemental Table 2. Background genome analysis of the seven Saga-*BPH* NILs (BC_5F_3) for BPH resistance with "Sagabiyori" genetic background

NIL	Resistance gene	Chr.	No. of polymorphic markers [a]	Range of donor segments (Mbp) [b]	Genome ratio (%) [c]	
					Donor	Sagabiyori
Saga-*BPH2*	*BPH2*	6	21	2.85 - 5.27	0.65	
		12	75	19.6 - 23.96	1.15	98.20
Saga-*BPH17ptb*	*BPH17ptb*	4	278	1.12 - 14.1	3.48	96.52
Saga-*BPH32*	*BPH32*	6	105	0.13 - 6.26	1.64	98.36
Saga-*BPH3*	*BPH3*	6	65	0.11 - 1.65	0.41	99.59
Saga-*BPH17*	*BPH17*	4	435	1.23 - 20.26	5.10	94.90
Saga-*BPH20*	*BPH20*	4	135	0.14 - 7.98	2.10	97.90
Saga-*BPH21*	*BPH21*	2	281	7.65 - 20.35	3.40	
		12	108	19.19 - 24.55	1.44	95.16

[a] Total polymorphic markers between the donor line and Sagabiyori

[b] Range of donor segments between polymorphic markers

[c] Genome ratio was calculated based on range of donor segments
